# Complete Genome Sequence of Cluster A1 Mycobacterium smegmatis Bacteriophage Arlo

**DOI:** 10.1128/MRA.01242-18

**Published:** 2018-10-25

**Authors:** Brittany Stewart, Megan Adams, Miranda Fuentes, Leeila Hanson, Esperanza Sandoval, Mario Tovar, Camille Trautman, Bianca Willis, Keith Emmert, Julie Edwards, Jesse Meik, James Pierce, Dustin Edwards

**Affiliations:** a Department of Biological Sciences, Tarleton State University, Stephenville, Texas, USA; b Department of Mathematics, Tarleton State University, Stephenville, Texas, USA; University of Maryland School of Medicine

## Abstract

Mycobacteriophage Arlo is a newly isolated *Siphoviridae* bacteriophage isolated from soil samples collected in Bluff Dale, Texas. Mycobacteriophage Arlo has a 52,960 base-pair double-stranded DNA genome that is predicted to contain 96 protein-coding genes. Mycobacteriophage Arlo is related to mycobacteriophage DD5 and other cluster A1 bacteriophages.

## Abstract (precis)

Mycobacteriophage Arlo is a newly isolated *Siphoviridae* bacteriophage isolated from soil samples collected in Bluff Dale, Texas. Mycobacteriophage Arlo has a 52,960 base-pair double-stranded DNA genome that is predicted to contain 96 protein-coding genes.

## ANNOUNCEMENT

**FIG 1:**
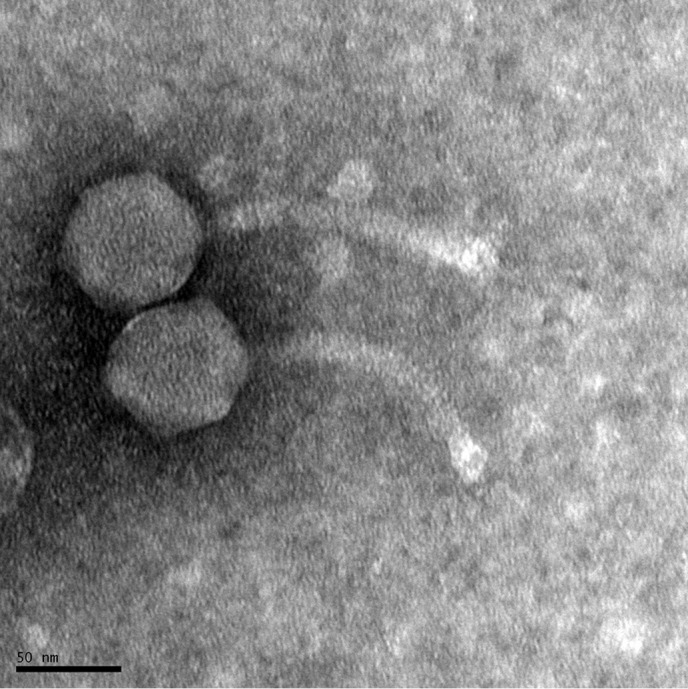
Transmission electron microscopy (TEM) of mycobacteriophage Arlo. Purified high-titer lysate was placed on a carbon type-B 300 mesh grid, stained with uranyl acetate, and imaged with an FEI Tecnai G^2^ Spirit BioTWIN transmission electron microscope (NL1.160G). TEM micrographs of negatively stained mycobacteriophage Arlo show an approximately 60-nm-diameter nonenveloped icosahedral capsid and 125-nm flexible noncontractile tail. The morphology of mycobacteriophage Arlo corresponds to that of members of the *Siphoviridae* family.

We report the whole-genome sequence of mycobacteriophage Arlo (1, 2), which was directly isolated from a strain of Mycobacterium smegmatis, a rapidly growing environmental species that is generally nonpathogenic but can act as an opportunistic pathogen in immunosuppressed individuals (3). Mycobacteriophage Arlo was isolated from compost-containing community vegetable garden soil samples collected in Bluff Dale, Texas (32°19′08.0004″, -098°01′14.9016″). Soil samples were washed with 7H9 liquid medium, and bacteriophages were extracted from the mixture through a 0.22-µm filter. For virus replication, filtered medium was incubated with Mycobacterium smegmatis mc^2^155 at 37°C for 48 h. Plaque assays of isolated mycobacteriophage Arlo resulted in medium-sized turbid plaques. Negative-staining transmission electron microscopy showed that mycobacteriophage Arlo has a siphoviral morphology with a 60-nm-diameter nonenveloped icosahedral capsid and a 125 nm flexible noncontractile tail, which is typical of viruses in the *Caudovirales* order (Fig. 1).

DNA was isolated from purified bacteriophage with the Promega Wizard DNA clean-up kit, and sequencing libraries were prepared from genomic DNA with the NEBNext Ultra II kit. Libraries were sequenced with Illumina MiSeq at the Pittsburgh Bacteriophage Institute to approximately 1,987-fold coverage from 742,500 total reads of 150-base read length (4). Sequence reads were assembled with Newbler 2.9 with default settings to produce a single-bacteriophage contig, which was checked for completeness, accuracy, and genome termini using *consed* v29.0 (5). The virus was determined to contain a linear double-stranded DNA genome that is 52,960 base pairs in length, with 63.8% G+C content, and a 3′ single-stranded terminal overhang of 5′-CGGATGGTAA-3′. Whole-genome nucleotide alignment with NCBI BLASTn (https://blast.ncbi.nlm.nih.gov/) (6) showed 96–97% nucleotide identity to cluster A1 mycobacteriophages Oogway (GenBank accession number MH230878) and DD5 (GenBank accession number NC_011022) (2).

Autoannotation of the genome was performed using GLIMMER v3.02 (7, 8) and GeneMark v2.5p (9, 10), followed by manual inspection, refinement of start sites, and annotation revision using Phamerator (https://phamerator.org/) (11), DNA Master v5.23.2 (http://phagesdb.org/DNAMaster/), and PECAAN (https://pecaan.kbrinsgd.org/). Mycobacteriophage Arlo is predicted to contain 96 protein-coding genes. No tRNAs genes were identified by ARAGORN v1.2.38 (12) or tRNAscan-SE v2.0 (13). Start codon usage was determined to be 90.12% AUG, 8.72% GUG, and 1.16% UUG.

HHpred v3.0beta (14, 15) and NCBI BLASTp (6) software were used to assign putative functions to 34 (35.4%) of 96 predicted protein-coding genes. The mycobacteriophage Arlo genome is arranged with rightwards-transcribed genes (genes 1 to 36, 58.4% of genome) encoding virion structural and assembly proteins and a lysis cassette consisting of lysin A and lysin B genes. Leftwards-transcribed genes encode DNA polymerase I, metallophosphoesterase, DNA primase, DNA methylase, endonuclease VII, NrdH-like glutaredoxin, DnaB-like dsDNA helicase, RecB-like exonuclease/helicase, and immunity repressor proteins.

### Data availability.

The mycobacteriophage Arlo genome is available at GenBank as accession number MH576971. Raw reads are available in the SRA under accession number SRX4721440.
